# Quality of Life and the Role of Gender in Patients With Non-Melanoma Skin Cancer in Greece

**DOI:** 10.7759/cureus.77195

**Published:** 2025-01-09

**Authors:** Nikolitsa S Dareioti, Christos Τ Lampropoulos, Balasis B Stavros, Sophia Georgiou, Philippos Gourzis, Nicholas S Mastronikolis

**Affiliations:** 1 Department of Dermatology, Patras University Hospital, Patras, GRC; 2 Department of Business Administration, University of Patras, Patras, GRC; 3 Department of Plastic Surgery, Patras University Hospital, Patras, GRC; 4 Department of Psychiatry, Patras University Hospital, Patras, GRC; 5 Department of Otolaryngology, Patras University Hospital, Patras, GRC

**Keywords:** basal cell cancer, female population, gender, quality of life, skin cancer, squamous cell cancer

## Abstract

Background

Improving quality of life (QoL), is an important factor for patients diagnosed with non-melanoma cell cancer (NMSC). NMSC is the most common and widespread cancer in the European population.

Methods

To measure QoL, several tools have been created, which try to capture and evaluate it both before and after treatment. The factors that contribute to the evaluation and the degree of impact on the patients' QoL are numerous and sometimes overlap or interact with each other. In the present study, the questionnaire used was the General Health Questionnaire-28 (GHQ-28).

Aims and objectives

The aim of this study is to map and highlight the population groups whose QoL is affected to a greater extent than others. The final sample of the population consisted of 103 patients (63 men and 40 women) with proven NMSC (via biopsy), who presented to the Dermatology Clinic of the University General Hospital of Patras during the period 2022-2023.

Results

Of the final study sample, 72 (69.9%) patients had one tumor, 23 (22.3%) patients had two tumors, 6 (5.8%) patients had three tumors, and 2 (1.9%) patients had more than three tumors. In 74.8%, the diameter of the tumor was less than 2 cm, while in 25.2%, it was greater than or equal to 2 cm.

Conclusion

Our results showed that, in general, the QoL of the female population, in all its aspects (psychology, bodily functions, appearance, etc.), is more affected compared to the corresponding male population, in cases diagnosed with NMSC. The effect may be due to multifaceted factors, such as the outcome of the treatment, the size of the tumor itself, aesthetic interventions, and the change in the patient's daily activities.

## Introduction

Basal cell carcinoma (BCC) and squamous cell carcinoma (SCC), the main types of non-melanoma skin cancer (NMSC), are the most common and widespread skin cancer in the European population [1]. Nevertheless, the precise incidence of BCC and SCC remains unknown [2]. The highest incidence rates have been reported in Australia (mean incidence of 1000/100,000 inhabitants), the USA (mean incidence of 212-407/100,000 inhabitants), and Europe (mean incidence of 76.21/100,000 inhabitants per year) [3].

Various risk factors such as unprotected exposure to ultraviolet (UV) radiation from the sun or solarium, weakened immune system due to illness or certain immunosuppressive drugs, history of skin cancer, fair skin, and gender may contribute in different ways to skin cancer. Each of these risk factors can contribute to skin cancer in different ways. Therefore, the combination of these factors not only affects the individual patient but also influences the specific type of skin cancer that develops [4]. Thus, it is urgently necessary to highlight the importance of accurate knowledge to facilitate appropriate early diagnosis and treatment to protect potential and already diagnosed patients from the dangers of skin cancer [5]. The lack of standardized reporting protocols with defined criteria for diagnostic and prognostic information may lead to incorrect recording of key information. This usually causes devastating adverse effects for the patient, as well as a lost opportunity to collect, sort, and analyze data for audit and research [6].

Improving quality of life (QoL) is an important factor for those patients diagnosed with NMSC [7]. To measure QoL, several tools have been created, which try to capture and evaluate it both before and after treatment [8]. Some of them are general, for use in the wide population, and can be applied to various conditions, while others are disease-specific and related to a specific pathology. However, it is important to note that QoL may be experienced differently and include different values within and between different cultural groups and populations within a country [9].

The aim of this study is to map and highlight these population of gender groups, whose QoL is affected to a greater extent due to the occurrence of NMSC compared to others.

## Materials and methods

Setting and analyzing population

The initial sample consisted of 147 patients (85 men and 62 women) with proven NMSC (via biopsy) who presented to the Dermatology Clinic of the General Hospital of Patras during the period 2022-2023. In all patients, the consent form was distributed and then collected for the research file, respecting all protocols and existing legislation for the protection of personal data. All the patients who participated in the survey were residents of the Region of Western Greece. The inclusion in the process of completing the questionnaire and in general the research lasted for each of them for a period of eight to nine months. A total of 39 people did not wish to continue in the repeated three-month filling or in the repeated six-month filling of the questionnaire. Five people did not fill the questionnaires in the appropriate way (they left many questions unanswered). The final sample of the population consisted of 103 patients (63 men and 40 women), who completed their participation in the research successfully (by completing the questionnaire before treatment, after three months of treatment, and after six months of treatment.

Sample characteristics

The age of the sample ranged from 21 years to 98 years, with an average of 67.79 years. The largest percentage of the sample (47.6%) were married, and 65% were retired. Regarding education, 26.2% of the sample had received post-secondary education. The largest percentage of patients had one tumor, with a 66% occurrence rate, on their face; 74.8% of the tumors had a diameter of less than 2cm. Overall, 58.3% of the sample was affected by BCC, 32% by SCC, and 9.7% by both. Of the sample, 82.5% underwent surgery, 1.1% were given cryotherapy, 2.6% were given radiotherapy, and 0.2% were given drugs (e.g., 5-fluorouracil) (Table 1). It is noteworthy that 70.9% of the sample was receiving medication (21.4% were receiving antidepressants and anxiolytics), and, in particular, the female population was receiving antidepressants and anxiolytics at a rate of 37.5% of all women who participated in the research.

**Table 1:** Demographic, socioeconomic, and clinical characteristics of patients.

Characteristic	Category/value	n (%)	p-Value
Age (years)	Mean (range)	67.79 ( 21-98 years)	˂0.001
Duration of disease	Mean (range)	4.01 (1-12 months)	˂0.001
Sex	Male	63 (61.2%)	˂0.001
	Female	40 (38.8%)	
Marital status	Single	12 (11.7%)	˂0.01
	Divorced	19 (18.4%)	
	Married	49 (47.6%)	
	Separated	10 (9.7%)	
	Widow/widower	13 (11.8%)	
Education level	Illiterate	28 (27.2%)	˂0.001
	Gymnasium	23 (22.3%)	
	High school	25 (24.3%)	
	Bachelor’s degree	20 (19.4%)	
	Master’s degree	4 (3.9%)	
	Doctorate degree	3 (2.9%)	
Professional status	Unemployed	6 (5.8%)	˂0.001
	Employee	30 (29.1%)	
	Retired	67 (65%)	
Annual income	Up to €5,000	19 (18.4%)	˂0.001
	Up to €10,000	28 (27.2%)	
	Up to €15,000	25 (24.3%)	
	Up to €20,000	16 (15.5%)	
	Up to €25,000	8 (7.8%)	
	Up to €30,000	2 (1.9%)	
	Above €35,000	5 (4.9%)	
Previous history of therapy	Yes	20 (19.4%)	˂0.001
	No	77 (74.8%)	
	Unknown	6 (5.8%)	
Type of skin cancer	Basal cell carcinoma	60 (58.3%)	˂0.001

Methods

To measure patients' QoL, several tools have been created that attempt to capture and evaluate it both before and after treatment. Some of them are general, for use in the general population, and can be applied in various situations, while others are disease-specific and related to a specific pathology [10].

The reason for choosing the General Health Questionnaire-28 (GHQ-28) is that it has been globally ranked as the most appropriate tool that measures emotional distress, emotional stress, and psychosocial interference with QoL within a clinical setting. It includes four subscales: i) somatic symptoms, ii) anxiety and insomnia, iii) social dysfunction, and iv) severe depression. The scale includes 28 questions, which are answered using a Likert-type 4-point rating scale: 0, 1, 2, and 3. [11]. Goldberg et al. suggest that patient participants in ongoing research studies who score 23 or less should be categorized as non-psychiatric, and patient participants with a score greater than 24 should be categorized as psychiatric. This is certainly not a panacea nor an element that should be considered in absolute terms [12]. Specifically, the GHQ-28 questionnaire asks patient participants to describe their “health profile” over the past period of time using behavioral items on a 4-point scale indicating the following frequencies of experience: “not at all,” “no more than usual,” “rather more than usual,” and “much more than usual” [13].

Like most questionnaires, they are divided into two main parts. The first part focuses on general questions that primarily describe the demographic and personal data of the patients. The latter were assisted by their dermatologist in completing this section of the questionnaire. These questions cover age, gender, economic status, number and size of tumors, educational level, duration of the disease, type of skin cancer, profession, and method of treatment. The second part includes questions from the General Health Questionnaire (GHQ-28), which measure quality of life, and are completed by the patients themselves [14].

Statistics tools

The statistical tool used was SPSS Version 29 (IBM Corp., Armonk, NY). The collected data were analyzed using descriptive statistics, including measures such as the mean and standard deviation. Subsequently, t-test methodologies were applied to compare mean scores, as well as Pearson correlations, to calculate and evaluate the different styles with QoL. Continuous variables were described as mean and standard deviation. For the comparison of continuous variables as well as independent variables, we used an independent t-test for normally distributed data. In addition, in contrasting cases, the ANOVA methodology was used. As mentioned, the correlation between continuous variables was assessed using Pearson correlation coefficients, as mentioned previously, where a two-tailed p-value of <0.05 was considered statistically significant.

Additionally, very important data were extracted based on the ANOVA method, where, for example, a correlation analysis (one-way ANOVA) was performed on the variable "sex" and the "mean" of the GHQ-28 with the following data: i) "before treatment" (F = 4.286; p = 0.041), ii) "after 3 months" (F = 5.096; p = 0.026, and iii) "after 6 months" (F = 4.843; p = 0.030).

Cronbach’s alpha is a measure of the internal consistency or reliability between several different items, measurements, or ratings. For questionnaire reliability testing, Cronbach’s alpha measures the internal consistency of a questionnaire or at least the domain(s) of a questionnaire. As is known, the values in Cronbach's alpha can range from 0 to 1. It is well-known that Cronbach's alpha values range from 0 to 1. The closer these values are to 1, the more reliable or internally consistent the items are in measuring the same variable or dimension, indicating that the questionnaire exhibits reliability or internal consistency. Conversely, when Cronbach's alpha values approach 0, it means that some or all of the items do not measure the same dimension, and the questionnaire does not exhibit reliability or internal consistency [15]. In our research, the Cronbach alpha of GHQ-28 was 0.928 before treatment, 0.934 after three months of treatment, and 0.944 after six months of treatment, values which indicate great reliability.

## Results

A total of 103 patients with NMSC (63 men and 40 women), aged between 21 and 98 years (M = 67.79, SD = 15.36), participated in this study. Of the 103 patients, 60 had BCC, 33 had SCC, and 10 patients had both SCC and BCC. In all four subcategories of the GHQ-28 and in the three patients’ response phases (before treatment, after three months of treatment, and after six months of treatment), the mean of the responses between the male and female samples was greater for the female population (Table 2).

**Table 2:** Analysis of means of the sub-categories of GHQ-28 per gender.

Questionnaire GHQ-28	Sample	Mean before treatment		Mean after three months		Mean after six months		Percentage difference for mean before treatment and after six months
		Mean before treatment	Percentage difference	Mean before treatment	Percentage difference	Mean before treatment	Percentage difference	
Somatic symptoms	Men	0.96	+29.16%	0.94	+32.98%	0.88	+39.77%	-8.33%
	Women	1.24		1.25		1.23		-0.81%
Anxiety and insomnia	Men	0.98	+21.43	0.89	+21.35%	0.86	+23.25%	-12.24%
	Women	1.19		1.08		1.06		-10.92%
Social dysfunction	Men	1.04	+6.73	0.91	+8.08%	0.87	+6.89%	-16.35%
	Women	1.11		0.99		0.93		-16.22%
Severe depression	Men	0.26	+53.85%	0.22	+81.81%	0.22	+77.27%	-15.38%
	Women	0.40		0.40		0.39		-2.5%

As shown in Table 2, the biggest difference between the male and female population was in the "severe depression" category, where the percentage of women compared to men ranged from +53.85% to +81.81%. This suggests that in our sample, the occurrence of NMSC affected the female population more in terms of psychology and depression. Similar results have been reported in a 2024 study conducted by Hora et al. in 182 patients [16].

Another very important element is that the category that showed the greatest recovery six months after treatment was "social dysfunction," with an improvement rate of 16.35% for men and 16.22% for women.

The smallest indicators of improvement in the QoL after six months appeared in the female population, particularly in the "somatic symptoms" category, where the average decreased by only 0.81%. In the same category, the male population also had the lowest percentages since the average decreased by 8.33%. The category in which patients had the highest average before treatment (2.2), after three months of treatment (2.19), and after six months of treatment (2.11) was "somatic symptoms." Therefore, the average of responses ranged from “worse than usual” to “much worse than usual.”

The category in which patients had the lowest average before treatment (0.66), after three months of treatment (0.62), and after six months of treatment (0.61) was "severe depression."

## Discussion

NMSCs are the most prevalent type of cancer among the Caucasian population worldwide, with SCC and BCC accounting for around 95% of them. Consequences of skin tumors and treatment modalities with resulting scars and deformities may lead to psychological and physical disorders [14,17].

From a psychological perspective, a patient's coping with skin cancer is not a simple process or one that follows specific norms and methodologies. Rather, it is a continuous process that can change and each time the patient follows different paths, pathways, and strategies that are mainly based on emotion [18]. According to several studies, when tumors appear on parts of the body that are visible to the public, especially in the facial areas, they cause behavioral, emotional, and cognitive levels with increased psychological morbidity [19]. On average, the female population with NMSC was more affected than the male population. This was particularly evident in the "somatic symptoms" category, where women were affected by 8.54% more, and in the "social dysfunction" category, where they were affected 1.5% less. In particular, six months after the therapeutic intervention, the indicators of the QoL of the male population improved by 13.30% compared to the period of the pre-treatment diagnosis, while the corresponding ones of the female population improved by 9.33%. In a prospective study of 183 patients with NMSC, women, regardless of lesion location, were significantly more likely to have lower QOL at four months post-treatment [20].

One reason may be that 82.5% of the cases were treated with surgery. Another reason is that 89.3% of the tumors had appeared in visible areas of the body (neck, face, and head), which have been reported to affect both the aesthetics and psychology of female patients more significantly [21-23]. A third reason could be the fact that the female majority of the sample, at a rate of 37.5%, was already receiving medication (antidepressants/sedatives), which may have had a greater impact on the burdened psychology of the female patients.

Limitations

As mentioned earlier, the initial sample consisted of 147 patients (85 men and 62 women) with proven NMSC (via biopsy) who presented to the Dermatology Clinic of the General Hospital of Patras during the period 2022-2023. Although those initially interviewed expressed a willingness to participate in the study, the geographical distance between their place of residence and the Dermatology Clinic of the General Hospital of Patras made their participation difficult. As a result, they later decided not to continue completing the follow-up questionnaires (after three and six months, respectively).

Another limitation is that approximately 10% of the participants were unable to understand the meaning of several terms/words in the questionnaire questions, resulting in the need for explanation and further analysis. Finally, as has been reported in other similar studies [14], more than half of the participants needed education and information to improve their knowledge about the various aspects of the disease they had been diagnosed with, as well as the degree of risk and mortality. Information and education on these issues will help in the future to have a more objective approach to emerging diseases, such as NMSC, on the part of patients, which will help improve their QoL.

## Conclusions

In conclusion, the results of the GHQ-28 questionnaire indicated that patients diagnosed with NMSC experienced a decline in QoL, with the female population being the most significantly affected. This population after six months of treatment was also the one that could not recover faster compared to their male counterparts.

This is of great value since both dermatologists and psychologists should be aware of the alteration of the QoL, especially in female patients with skin cancer, to be able to support and inform them both before and after treatment. Lastly, these results could lead to specialized interventions or to the creation of specific handbooks to provide psychological support tailored to women.
